# Deep tissue sequencing improves genetic diagnostic yield in focal cortical dysplasia^☆^

**DOI:** 10.1016/j.pnpbp.2026.111920

**Published:** 2026-09-06

**Authors:** Breana Galea, Joshua Reid, Samuel Gooley, Tom Witkowski, Tara Lane, Sian Macdonald, Timothy E. Green, Zimeng Ye, Thiuni Adikari, Kristian Bulluss, Saul A. Mullen, Caitlin A. Bennett, Brialie Forster, Gabi Bradshaw, Wendi Lin, Wasanthi De Silva, Rosita B. Ramirez, Sattar Khoshkhoo, Sachin Gupta, Michael Krivanek, Kavitha Kothur, Deepak Gill, Kate Pope, Greta Gillies, Matthew Coleman, Wei-Shern Lee, Sarah M. Stephenson, Wirginia Maixner, A. Simon Harvey, Emma Macdonald-Laurs, Katherine B. Howell, Colleen D’Arcy, Paul J. Lockhart, Richard J. Leventer, Renata M. Kalnins, Jonathan Clark, Mark F. Bennett, Melanie Bahlo, Ingrid E. Scheffer, Piero Perucca, Samuel F. Berkovic, Michael S. Hildebrand

**Affiliations:** a Epilepsy Research Centre, Department of Medicine, The University of Melbourne, Austin Health, Heidelberg, Victoria 3084, Australia; b Florey Institute of Neuroscience and Mental Health, The University of Melbourne, Parkville, Victoria 3052, Australia; c Genetics and Gene Regulation Division, The Walter and Eliza Hall Institute of Medical Research, Parkville, Victoria 3052, Australia; d Department of Medical Biology, The University of Melbourne, Parkville, Victoria 3052, Australia; e Murdoch Children's Research Institute, The Royal Children's Hospital, Parkville, Victoria 3052, Australia; f Department of Neurosurgery, Austin Health, Heidelberg, Victoria 3084, Australia; g Molecular Pathology, Austin Health, Heidelberg, Victoria 3084, Australia; h Department of Neurology, Brigham and Women's Hospital, Harvard Medical School, Boston, MA 02115, USA; i Division of Genetics and Genomics, Manton Center for Orphan Disease Research, Boston, MA 02115, USA; j Broad Institute of Massachusetts Institute of Technology and Harvard, Cambridge, MA 02142, USA; k TY Nelson Department of Neurology and Neurosurgery, The Children's Hospital at Westmead, Sydney, New South Wales 2145, Australia; l Department of Histopathology, The Children's Hospital at Westmead, Sydney, New South Wales 2145, Australia; m University of Sydney, The Children's Hospital at Westmead, Sydney, New South Wales 2145, Australia; n Kids Neuroscience Centre, The Children's Hospital at Westmead, Sydney, New South Wales 2145, Australia; o Department of Paediatrics, The University of Melbourne, The Royal Children's Hospital, Parkville, Victoria 3052, Australia; p Department of Neurosurgery, The Royal Children's Hospital, Parkville, Victoria 3052, Australia; q Department of Neurology, The Royal Children's Hospital, Parkville, Victoria 3052, Australia; r Department of Anatomical Pathology, The Royal Children's Hospital, Parkville, Victoria 3052, Australia; s Anatomical Pathology, Austin Health, Heidelberg, Victoria 3084, Australia

**Keywords:** Dysplasia, Epilepsy, Epilepsy surgery, Lesion, Mosaicism, Sequencing

## Abstract

Focal cortical dysplasias (FCDs) are malformations of cortical development associated with drug-resistant focal epilepsy. We analyzed surgical tissue from 25 consecutive cases recruited from adult and pediatric epilepsy surgery programs. We performed high-depth sequencing of lesional tissue, validated somatic variants using droplet digital PCR or amplicon sequencing, and investigated genotype-phenotype correlations. A pathogenic or likely pathogenic variant was detected in 64% (*n* = 16/25) of cases. Of these, five cases with FCDIIa or FCDIIb had germline variants in *NPRL3* (*n* = 3) or *DEPDC5* (*n* = 2). Somatic variants were identified in 44% (*n* = 11/25) of cases. The genetic yield for FCDIIb was 77% of cases having a pathogenic mTOR pathway variant detected (*n* = 10/13), and for FCDIIa 66% (*n* = 6/9). High depth sequencing approaches allowed detection of somatic variants with very low (down to 0.4%) variant allele fractions (VAFs). No pathogenic variants were detected in 3 cases with FCDI. 62% (*n* = 15/24) of the cases with ≥12 months follow up experienced a favourable seizure outcome (Engel 1–2) following surgery. Of note, *n* = 9 patients required repeat surgery to resect residual dysplasia. Determining a genetic diagnosis reveals aetiology and paves the way to precision therapies that may benefit those with FCD who do not respond to current treatments.

## 1. Introduction

Focal cortical dysplasia (FCD) is the most common histopathological diagnosis amongst children, and second most common amongst adults, with drug-resistant focal epilepsy requiring surgery (Blümcke et al., 2017). For these patients seizure freedom is often only achievable through surgical resection and in a subset a second surgery is required to remove residual dysplasia (Roessler et al., 2018). FCD comprises distinct subtypes with characteristic pathological and imaging features. FCDI is characterised by abnormal cortical lamination and lesions are often small and hard to detect radiologically. FCDIIa is characterised by cortical dyslamination with dysmorphic neurons, as is FCDIIb along with balloon cells; both typically show cortical thickening and grey–white blurring with or without a transmantle sign on MRI. FCDIII combines cortical dyslamination with an adjacent lesion (Blümcke et al., 2011).

The major genetic etiologies of FCD are germline and somatic pathogenic variants of genes in the mechanistic (formerly mammalian) target of rapamycin (mTOR) pathway (Perucca et al., 2022; Lim et al., 2014; D'Gama et al., 2017; Marsan and Baulac, 2018; Baldassari et al., 2019a, 2019b; Najm et al., 2022; Ferri et al., 2024; Macdonald-Laurs et al., 2025). Reduced penetrance of some germline variants in these genes and case reports of biallelic variants supports a two-hit genetic model, similar to neoplasms, where inherited germline variants confer risk and interact with a second, somatic variant in brain tissue to initiate development of a dysplastic cortical malformation (Bennett et al., 2022; D'Gama et al., 2017; Lee et al., 2019; Ribierre et al., 2018).

Genetic testing for FCD typically involves germline testing of blood-derived DNA via exome sequencing (ES) or gene panels targeting mTOR pathway genes. For somatic testing of resected brain tissue high depth sequencing is often needed to detect pathogenic variants at variant allele fractions below 5%, which are now known to account for many cases of FCDII (Baldassari et al., 2019b). Targeted gene panels that allow deep sequencing at ≥1000× depth are often necessary to detect these low-level somatic variants. In the research setting, single cell genotyping and transcriptomics applied to surgical FCD tissue have revealed that mTOR mutations are present in multiple cell types with cell-type specific transcriptional dysregulation occurring in both mutated and non-mutated cells (Baldassari et al., 2025; Bizzotto et al., 2025). Such approaches hold promise for more precise future diagnostic and therapeutic stratification.

Across surgical cohorts, FCD subtype is one of the strongest predictors of postoperative seizure outcome. Consistently, patients with FCD type II, and particularly FCD type IIb, have better postoperative seizure outcomes than FCD type I (Tassi et al., 2002; Krsek et al., 2009; Lamberink et al., 2020). Several studies have explored epilepsy surgery outcomes in patients with FCD and a genetic diagnosis. In patients with FCD and a pathogenic germline mTOR variant, around 60% have a favourable seizure outcome after epilepsy surgery (Baldassari et al., 2019a, 2019b; Sanders et al., 2019; Lee et al., 2020; Boßelmann et al., 2022). In patients with FCD type II and a pathogenic somatic mTOR variant, up to 80% achieve seizure freedom after surgery (Baldassari et al., 2019b; Lee et al., 2021; Moloney et al., 2022). Whilst these cohorts are relatively small, the seizure outcomes are broadly consistent with those reported across different FCD subtypes in larger cohorts where genetic diagnosis was not included (Lamberink et al., 2020). Moreover, Macdonald-Laurs et al. (2024b) found a similar rate of favourable seizure outcomes after surgery in FCD patients who had a germline mTOR variant (90%) compared to those in whom no variant was detected (88%). Together these studies suggest that genetic diagnosis itself does not impact surgical outcomes in FCD.

In contrast, for patients with other types of epilepsy due to mutations in ion channel genes or genes involved in synaptic transmission, surgery is rarely effective (Stevelink et al., 2018). For example, of 17 patients with germline mutations in ion channel or synaptic transmission genes in one study, 16 were considered inappropriate for resective surgery, and the one patient who did undergo resective surgery was not seizure-free (Sanders et al., 2019).

Here, we aimed to (i) improve the genetic diagnostic yield for patients with FCD and drug-resistant focal epilepsy requiring surgery by interrogating deep tissue sequencing data for somatic variants, and (ii) determine the impact of a genetic diagnosis on seizure outcomes post-surgery.

## 2. Materials & methods

### 2.1. Ethical statemente

Our cohort comprised 25 cases with drug-resistant focal epilepsy where histopathology of surgically resected brain tissue confirmed FCD (see Supplementary Table 1 for patient list). Cases were recruited from three epilepsy surgery programs. Written informed consent was obtained for all cases with approval from Human Research Ethics Committees at Austin Health, Melbourne, The Royal Children's Hospital, Melbourne, The Westmead Children's Hospital, Sydney and The Walter and Eliza Hall Institute of Medical Research, Melbourne.

Surgically resected fresh-frozen (FF) or formalin-fixed paraffin-embedded (FFPE) brain tissue was collected from all cases. For further analysis of variants, whole blood and buccal samples were also collected where possible. Genomic DNA was extracted using commercially available kits, including the QIAamp DNA Blood Maxi Kit for whole blood and DNA Genotek prepIT.L2P for buccal samples. The QIAamp DNA FFPE Tissue Kit was used to extract FFPE brain specimens. The same kit was used to extract DNA from fresh frozen brain specimens with the deparaffinisation step removed from the protocol.

### 2.2. Exome sequencing

DNA libraries were prepared using the SureSelect Human All Exon V6 Kit (Agilent) and sequenced on either an Illumina NovaSeq 6000 or X Plus system at BGI Genomics (Hong Kong) with 150 bp paired-end reads, targeting either 80×, 200×, 400× or 1000× exome-wide coverage per sample (Supplementary table 1). Read alignment to the GRCh38 human reference genome was performed using BWA-MEM, followed by realignment and recalibration with GATK. Variant calling was performed using the Sarek pipeline from Nextflow Core (Garcia et al., 2020), which implements multiple somatic callers; Mutect2, Strelka2 and FreeBayes.

### 2.3. High-depth targeted panel sequencing

DNA libraries were prepared using the SureSelect XT HS2 DNA Kit (Agilent) with duplex unique molecular identifiers (UMIs). Two gene panels were used, Panel 1 targeted 140 genes including 36 Ras/Raf/MAPK pathway genes, 12 mTOR pathway genes, 7 other focal epilepsy genes and 85 additional cancer genes (see Supplementary Table 2 for gene list) and Panel 2 targeted 71 genes including 43 Ras/Raf/MAPK pathway genes, 12 mTOR pathway genes and 16 other epilepsy genes (Supplementary Table 3 for gene list). Sequencing was performed on an Illumina NextSeq 1000 platform (Panel 1) and an Illumina NovaSeq 6000 platform (Panel 2) with 150 bp paired-end reads. Read alignment to the GRCh38 human reference genome and variant calling were performed using the DRAGEN DNA Pipeline on Illumina Basespace (v4.3.13). UMI tags were used for read collapsing during alignment, achieving a median consensus depth of 582× per sample across Panel 1 and an average consensus depth of 1835× on Panel 2.

### 2.4. Variant prioritisation

Variant analysis of ES data was restricted to genes in a curated list of 1371 genes derived from: Genes4Epilepsy (v2025–03, Oliver et al., 2023), KEGG Ras and mTOR signalling pathways (Kanehisa et al., 2025; Kanehisa, 2019; Kanehisa and Goto, 2000), PanelApp Australia Malformations of Cortical Development Superpanel (v4.65, Martin et al., 2019), and the genes on Panels 1 & 2. Variants in all coding exons were filtered and were required to have ≥2 alternate reads on each strand (forward and reverse). For the targeted gene panels (Panels 1& 2) all coding exons were filtered unless there were known hot spots, in which case only coding exons in those regions were filtered and each variant required at least 2 supporting duplex consensus reads.

Variants were annotated using Ensembl v115.2, GRCh38 Variant Effect Predictor and filtered using the following tools: 1) Variants with gnomAD v4.0.0 population allele frequency > 0.1% were excluded. 2) Variants with CADD v1.6 pathogenicity scores <20 were excluded. 3) Variants classified as benign or likely benign in ClinVar (ClinVar release on 30th March 2025) were excluded. Variants were additionally reviewed using Franklin by Genoox (Qiagen, Hilden, Germany, website accessed between March and November 2025) to aid pathogenicity classification. As Franklin applies ACMG/AMP criteria developed for germline variant interpretation, pathogenicity assignments were considered supplementary and interpreted accordingly in the context of somatic variant analysis. Candidate variants were visualised in Integrative Genomics Viewer (IGV) v2.19.1 for manual quality assessment.

### 2.5. Droplet digital PCR and amplicon sequencing

Low frequency somatic variants (VAF < 6%) were validated utilising Bio-Rad droplet digital PCR (ddPCR) or amplicon sequencing. TaqMan^®^ SNP Genotyping Assays customed ordered from Thermo Fisher Scientific (Waltham, MA) were used for ddPCR. Fluorescently-labelled probes were added to ddPCR Supermix for probes (no dUTPs) (Bio-Rad, Hercules, CA) and mixed with 10 ng of DNA derived from surgically resected fresh-frozen brain tissue. Generation of droplets, PCR cycling and droplet reading were performed according to the manufacturer's instructions. Graphical outputs were created using RStudio. For amplicon validation, custom primers were designed using Primer3 default settings to generate 150-500 bp amplicons, synthesized by IDT, and tested on human genomic DNA (Promega) to confirm single-product amplification. Validated primers were applied to 5-20 ng of brain tissue gDNA using Phusion High-Fidelity PCR Master Mix (Thermo Fisher). Products were cleaned with 2× AMPure XP beads (Beckman Coulter), gel-verified, pooled, and Illumina sequenced to a minimum depth of 10,000 reads per amplicon.

### 2.6. Sanger sequencing

Germline and high frequency somatic variants were validated using PCR and Sanger sequencing. Gene variants were amplified using gene-specific primers (oligonucleotide sequences available on request) designed to the reference human gene transcripts (NCBI Gene). Amplification reactions were cycled using a standard protocol on a Veriti Thermal Cycler (Applied Biosystems, Carlsbad, CA). Bidirectional sequencing was completed with a BigDye^™^ v3.1 Terminator Cycle Sequencing Kit (Applied Biosystems), according to the manufacturer's instructions. Sequencing products were resolved using a 3730xl DNA Analyzer (Applied Biosystems). All sequencing chromatograms were compared to the published cDNA sequence; nucleotide changes were detected using Codon Code Aligner (CodonCode Corporation, Dedham, MA).

### 2.7. Immunostaining

Archival FFPE tissue sections from case 12 with a somatic *MTOR* variant were immunostained according to a published protocol (Baybis et al., 2004). Blocking buffer consisted of 2% fetal bovine serum (FBS) in 1× phosphate-buffered saline (PBS). FFPE tissue slides were immersed in citratebased antigen unmasking solution (#H-3300, Vector Laboratories, Newark, CA) and incubated overnight with Phospho-S6 Ribosomal Protein (Ser235/236) (D57.2.2E) XP^®^ Rabbit mAb (1:1000) (#4858, Cell Signalling Technology, Danvers, MA) at 4C. The slides were then incubated in secondary antibody Goat Anti-Rabbit IgG H&L (HRP) (1:1000) (#ab6721, Abcam, Cambridge, UK) for 1 h at room temperature. Immunoreactivity was visualised using the VECTASTAIN^®^ ABCHRP Kit, Peroxidase (Standard) (#PK-4000, Vector Laboratories) and DAB Substrate Kit, Peroxidase (HRP), with Nickel, (3,3′-diaminobenzidine) (#SK-4100, Vector Laboratories), counterstaining with hematoxylin for 10 s from the Hematoxylin and Eosin Stain Kit (#H-3502, Vector Laboratories). Commercial kits were used according to the manufacturer's recommendations.

### 2.8. Reverse transcription polymerase chain reaction (RT-PCR)

RNA was extracted from brain tissue or cultured fibroblasts using the Qiagen AllPrep DNA/RNA Kit. cDNA synthesis was performed with the SuperScript^™^ III First-Strand Synthesis System using random hexamer primers. Region-specific primers (Supplementary Table 4) were used to amplify cDNA by PCR. RT-PCR products were analyzed by gel electrophoresis and Sanger sequencing to assess variant impact on RNA splicing.

### 2.9. Phenotyping and statistical analysis

Seizure outcome was classified using the Engel scale (Engel Jr. et al., 1993). A single Engel class at last follow-up was assigned based on the postoperative seizure course from the most recent epilepsy surgery. An Engel class was only assigned if the patient had ≥12 months follow up from the date of their most recent resective surgery. A favourable seizure outcome was defined as Engel class 1 or 2 and an unfavourable seizure outcome was defined as Engel class 3 or 4.

In the phenotyping and genotype–phenotype correlation analyses, descriptive statistics were used to summarize clinical characteristics including age of seizure onset and age at first surgery. Comparisons between groups (e.g., germline vs. somatic variants, presence vs. absence of a pathogenic variant) were evaluated using Fisher's exact test for categorical outcomes such as surgical response, due to small sample sizes. For continuous variables like age of seizure onset, group comparisons were performed using the Mann–Whitney *U* test. Statistical significance was defined using a *p*-value threshold of <0.05.

## 3. Results

### 3.1. Clinical phenotypes

All patients (*n* = 25) underwent resective surgery for treatment of drug-resistant focal epilepsy. Focal cortical dysplasia was confirmed histopathologically in each case with the most common subtype being FCDIIb (*n* = 13 patients), followed by FCDIIa (*n* = 9) and FCDI (*n* = 3). The FCDs were most frequently located in the frontal lobe (*n* = 14), followed by the parietal lobe (*n* = 6), temporal lobe (*n* = 3), occipital lobe (*n* = 1) and a hemispheric dysplasia (*n* = 1); the latter case has previously been reported (Leventer et al., 2015). Of note, the FCD involved either the frontal or parietal cortex in 86% (*n* = 19/22) cases with FCDII (a or b). Across the cohort, the median age of seizure onset was 5 years (range: 6 weeks to 30 years), and the median age of first epilepsy surgery was 24 years (6 months to 63 years).

At least 12 months of post-operative follow up surgical outcome data (from the date of the most recent surgery) was available for *n* = 24 cases with a mean of 6.2 years of follow up (1–11 years). 62% (*n* = 15/24) experienced a favourable seizure outcome (Engel 1–2). Nine patients underwent repeat surgery to resect residual dysplasia. Of these 9 cases requiring repeat surgeries, five had a favourable seizure outcome after the repeat surgery and four had an unfavourable outcome. Patients with FCDIIb tended to have better surgical outcomes than those with FCDIIa or FCDI. Favourable seizure outcomes were seen in 83% of FCD IIb cases (*n* = 10/12), compared with 33% of FCDI cases (*n* = 1/3) and 44% of FCDIIa cases (*n* = 4/9). When FCDIIb was compared with both other FCD subtypes combined, this difference showed a nonsignificant trend (*p* = 0.089). However, these comparisons are underpowered due to small subgroup sizes.

### 3.2. Genetic findings

At least one pathogenic variant was detected in 64% (*n* = 16/25) of cases (Table 1). Germline pathogenic variants were identified in 20% (*n* = 5/25) of cases, comprising *NPRL3* (*n* = 3) and *DEPDC5* (*n* = 2). The *NPRL3* splice site variant (case 2) was shown to cause skipping of exon 5 in brain-derived RNA (see Supplementary Fig. 1). The germline *DEPDC5* variant in case 5 was part of a single nuclei RNA sequencing study of FCD that showed disruption of glutamate and GABA-A signalling pathways in variant-carrying neurons (Bizzotto et al., 2025). Pathogenic or likely pathogenic somatic *MTOR* variants were found in 44% (*n* = 11/25) of cases. Of the *n* = 11 cases with a pathogenic somatic variant, the median variant allele fraction (VAF) was 2% with a range of 0.4%–8.0%. All low-level variants (VAF <6%) were independently validated by either ddPCR or amplicon sequencing. Sequencing and validation VAFs showed moderate concordance (Pearson *r* = 0.726), with a mean absolute error of 0.72 percentage points and minimal systematic bias (mean bias −0.08 pp) (Supplementary Table 5).

In one FCDIIb case with a pathogenic somatic *MTOR* variant at a VAF of 5.4% (case 11), we were able to confirm hyperactivation of the mTOR pathway with immunostaining of lesional tissue demonstrating increased cytoplasmic phosphorylation of ribosomal S6 protein (p-S6) in dysmorphic neurons (Fig. 1). Somatic variants were absent in paired blood or buccal samples in the 10 variant-positive cases with paired samples available. Two (*n* = 2/25) double-hit germline-somatic cases were identified, one with a candidate germline variant in *EIF4ENIF1* and a pathogenic somatic *MTOR* variant (case 16), and one with a pathogenic germline *NPRL3* and a candidate somatic variant in *WNT2* (case 4). Case 3 is sibling of case 4 and also carries the germline *NPRL3* variant. We have previously reported this finding in cases 3 and 4 (Bennett et al., 2022) (Table 1).

### 3.3. Genotype–phenotype correlations

Pathogenic or likely pathogenic mTOR pathway variants were identified in 77% (*n* = 10/13) of cases with FCDIIb (1 germline, 9 somatic) and in 66% (*n* = 6/9) cases with FCDIIa (4 germline, 2 somatic). There was marked genetic homogeneity in the FCDIIb cases with a pathogenic somatic variant in the *MTOR* gene being detected in 69% (*n* = 9/13) of cases, compared to 22% (*n* = 2/9) of FCDIIa cases (Fig. 1). No pathogenic variants were identified in the three cases with FCDI despite examining all coding exons of *SLC35A2* (Winawer et al., 2018).

Of the *n* = 5 cases with a pathogenic or likely pathogenic germline mTOR pathway variant, the median age of seizure onset was 8 months old (range 3 months–21 years). This was earlier than the median age of 6 years (range 1.5 months–13 years) in the *n* = 11 cases with a somatic mTOR pathway variant. However, this difference was not statistically significant (*p* = 0.29) and the comparison is limited by small numbers in each group. The age of seizure onset across the cohort was broadly consistent with other reported FCD series (Rácz et al., 2018), with seizure onset occurring before the age of 18 years in the majority of cases (*n* = 22/25) with only a few cases (*n* = 3) having seizure onset in adulthood. These 3 cases with seizure onset in adulthood included a left frontal FCDIIb case with a germline *DEPDC5* variant, a right parietal FCDIIa case without a variant and a left occipital FCDIa case also with no variant detected. Patients with a germline variant tended to undergo surgery at a younger age (median of 6 years; range 2–49 years) compared to a median of 24 years (range 0.5–63 years) in those with a somatic variant. This difference was also not statistically significant (*p* = 0.27) and limited by small subgroup sizes.

Patients with a pathogenic variant (germline or somatic) had similar seizure outcomes after surgery compared to those without. Of those with a variant and > 12 months follow up, 67% (*n* = 10/15) had a favourable seizure outcome, compared to 55% (*n* = 5/9) with no variant detected. This difference was not statistically significant (*p* = 0.68) but the analysis is underpowered due to small subgroup sizes. Of the nine patients who underwent repeat surgeries, five had a pathogenic variant and four did not.

We were underpowered to explore whether variant allele fractions (VAFs) affected surgical outcomes in the *n* = 11 patients with a somatic *MTOR* variant and surgical follow up data available. However, similar VAFs were seen in those with favourable and unfavourable outcomes after surgery with VAFs ranging from 0.4 to 6.9% in those with a favourable surgical outcome and 1.2–8% in those with an unfavourable outcome. Median VAFs were 2.8% and 2.3%, respectively, with no significant difference between groups (*p* = 0.91).

## 4. Discussion

FCDIIb was the most common histopathological subtype, followed by FCDIIa, with the majority (86%, *n* = 19/22) of FCDII (a or b) being located in the frontal or parietal lobes, consistent with findings from other FCD cohorts (Macdonald-Laurs et al., 2024a, b). Those with a pathogenic germline mTOR pathway variant tended to have an earlier age of seizure onset compared to those with a somatic mTOR pathway variant, which is a phenomenon recently reported in patients with bottom-of-sulcus FCDs (Macdonald-Laurs et al., 2024a, b), although we were underpowered to confirm this difference was significant in our cohort. Patients with FCDIIb tended to have better surgical outcomes than those with FCDIIa or FCDI. We were also underpowered to show that this was a statistically significant difference in our cohort, but this phenomenon has been reported in larger cohorts (Tassi et al., 2002; Krsek et al., 2009; Lamberink et al., 2020).

We observed a higher rate of pathogenic somatic variant detection with a targeted gene panel approach that utilised high depth duplex sequencing than with exome sequencing (ES). ES was performed at varying depths (80×, 200×, 400× & 1000×; Supplementary Table 1), whereas the 2 targeted gene panels achieved median duplex consensus depths of 582× and 1500× respectively. Unlike raw reads, duplex consensus reads use UMI collapsing to eliminate PCR duplicates, ensuring each read reflects a truly independent molecular observation, conferring greater sensitivity. A somatic variant was detected in only 5/22 (23%) of patients with ES compared to 6/9 (67%) of patients who had a targeted gene panel. This included 4 patients (cases 6, 9, 13 & 16 Table 1) who had no variant detected on initial ES but subsequently had a low-level variant detected by the targeted gene panel approach. Moreover, all 5 patients who had a very low-level variant (VAF < 1.5%), had the variant detected using the high depth gene panel, this included variants with VAFs as low as 0.4% (Cases 6, 8, 9, 10 & 16 Table 1). This capacity to resolve low-level mosaicism resulted in a somatic variant being detected in 69% of our FCDIIb cases, which is a relatively high yield compared to most FCDII series that report somatic variants in ~35–60% of cases with median VAFs ~3–5% (Baldassari et al., 2019a, 2019b; Lai et al., 2022; Gerasimenko et al., 2023). This finding is consistent with recent studies that have shown that very-low VAF variants are an important cause of FCDII. For example, Kim et al., were able to increase their FCDII diagnostic yield to ~80% by preforming cell sorting to isolate p-S6–positive lesional cells prior to deep sequencing, which revealed mTOR pathway variants with VAFs <0.2% (Kim et al., 2023). Ultra-deep sequencing has since detected mosaicism down to 0.07% in bulk brain tissue from FCDII patients (Kim et al., 2024). Collectively, these data suggest that genetic testing for FCD should employ sequencing strategies that reliably detect variants with allele fractions below 1%.

The comparisons between sequencing methods in our study are observational only as this was a retrospective study that did not systematically compare methods head-to-head, and patients did not uniformly undergo all sequencing approaches (Supplementary Table 1). That said, our observations suggest that high-depth with duplex UMI error correction represents a practical first-line sequencing strategy for clinical genetic diagnosis in FCDII. For cases that remain negative after targeted panel sequencing, techniques such cell enrichment prior to sequencing (Kim et al., 2023) or ultra-deep amplicon sequencing (Kim et al., 2024) can solve previously negative cases; however, both face barriers to routine clinical implementation as they are more resource-intensive than standard sequencing approaches.

Double-hit germline-somatic variants in FCD are rarely reported and typically occur in the same gene (Lee et al., 2019; Ribierre et al., 2018). Notably, we detected double-hit germline-somatic variants in 2 cases (cases 4 and 16), each harbouring a single mTOR pathway variant alongside a candidate variant from an intersecting pathway (somatic *WNT2* or germline *EIF4ENIF1*). *WNT2* regulates mTOR indirectly via modulation of the TSC1/TSC2 complex (Inoki et al., 2006; Hermida et al., 2017), and we have previously shown that this *WNT2* variant causes partial loss of canonical WNT signalling (Bennett et al., 2022). *EIF4ENIF1* encodes a negative regulator of eIF4E (Dostie et al., 2000), a key downstream effector of the mTOR pathway implicated in the translational dysregulation underlying FCDII (Kim et al., 2019). Together, these findings raise the possibility that variants in intersecting pathways may act cooperatively to potentiate mTOR signalling and drive FCD pathology, although whether this reflects a distinct dualpathway mechanism or chance co-occurrence remains to be established.

In summary, the frequent detection of mTOR pathway gene variants in this study aligns with recent work in this field that has confirmed mTOR pathway dysregulation as the dominant cause of FCD. Recent advances in high-depth sequencing is enabling accurate detection of somatic variants at very low VAFs and cutting-edge single-cell genotyping and transcriptomic techniques are rapidly expanding our understanding of the developmental timing of these variants, the specific cell types involved and how these affected cells are interacting with surrounding brain tissue to produce seizures (Kim et al., 2024; Bizzotto et al., 2025; Baldassari et al., 2025). This integration of genetic diagnosis with mechanistic biology is paving the way for the emerging use of FDA-approved mTOR inhibitors like everolimus for individuals who are positive for an mTOR pathway gene variant and who continue to have seizures despite current treatments.

## Supplementary Material

1

## Figures and Tables

**Fig. 1.**
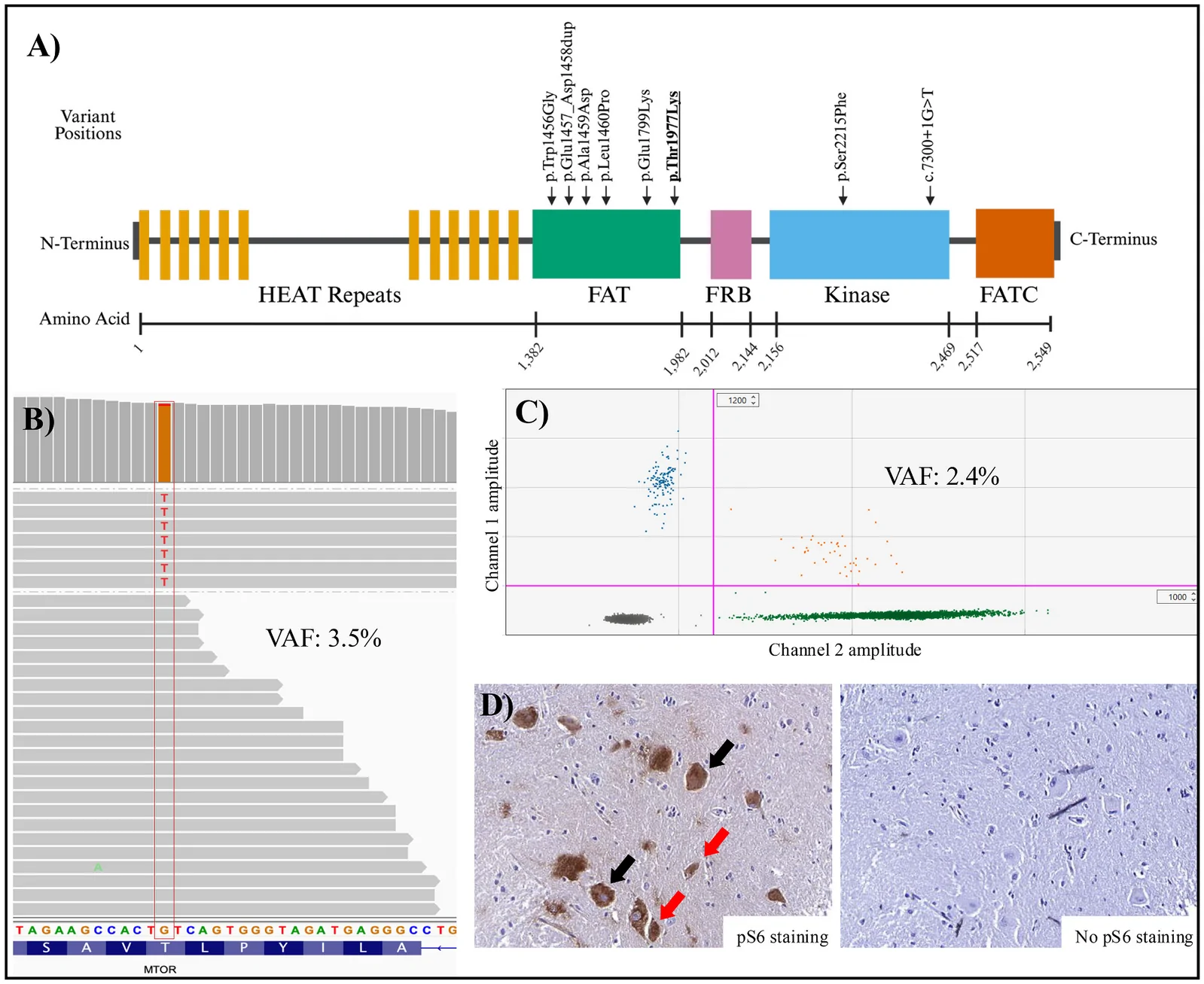
Molecular and functional results for low frequency MTOR variant (c.5930C > A, p.Thr1977Lys) found in genomic DNA of case 11 with FCD IIb. VAF: variant allele fraction. p-S6: 22hosphor-S6. Top-left: Diagram of MTOR variant. Created using BioRender. Top-right: Screenshot of exome data results in fresh-frozen brain tissue using Integrative Genomics Viewer. The MTOR variant is present at a VAF of 3.5% (7 reads in a total of 193 reads). The box indicates the position of the nucleotide change. Middle-left: Graph representing droplet digital PCR (ddPCR) validation in fresh-frozen brain tissue at a VAF of 2.4%, with the y-axis corresponding to channel 1 and the x-axis corresponding to channel 2. Results represent mutant alleles (blue droplets), wildtype alleles (green droplets), wildtype and mutant alleles (orange droplets) and no DNA template (grey droplets). Bottom: Two histology images showing FFPE tissue sections with or without p-S6 staining. Red arrows show dysmorphic neurons and black arrows show balloon cells. Images cropped from whole slide scans taken at 40× magnification on the PALM Laser Dissector System Axiovert 200 M (ZEISS, Oberkochen, Germany).

**Table 1:** Summary of findings in novel and established epilepsy genes.

Case.	Histopathology	Anatomical Location	Age at Surgery (years)	Surgical Outcome (Engel)	Gene Variant	VAF (%)
†1	FCDIIa	Right temporal	3	2a	*DEPDC5* (c.2390delA, p. Gln797Argfs*18)	Germline
2	FCDIIa	Left frontal (cingulate gyrus)	7	1a	*NPRL3* (c.393 + 4_393 + 7del)	Germline
†3	FCDIIa	Left mesial frontal	2	1a	*NPRL3* (c.48delG, p.Ser17Alafs*70)	Germline
†4	FCDIIa	Right superior frontal gyrus (posteriorly)	6	4a	*NPRL3* (c.48delG, p.Ser17Alafs*70) *WNT2* (c.338C > T, p.Ala113Val)	Germline 0.3 (Candidate)
†5	FCDIIb	Left frontal pole	49	1a	*DEPDC5* (c.4501C > T, p.Gln1501X)	Germline
6	FCDIIa	Left frontal	0.5	4	*MTOR* (c.6644C > T, p.Ser2215Phe)	1.2
†7	FCDIIa	Right hemispheric (extensive)	0.8, 3.8	3a	*MTOR* (c.4487 T > G, p.Trp1456Gly)	8.0
8	FCDIIb	Right temporal	63	2a	*MTOR* (c.5395G > A, p.Glu1799Lys)	0.4
9	FCDIIb	Left sup frontal gyrus	24	1a	*MTOR* (c.4379 T > C, p.Leu1460Pro)	0.7
10*	FCDIIb	Left frontal (precentral sulcus)	23	NA	*MTOR* (c.4376C > A, p.Ala1459Asp)	1.4
11	FCDIIb	Right parietal / precuneus	25	1b	*MTOR* (c.5930C > A, p.Thr1977Lys)	5.4
12	FCDIIb	Right parietal (post central sulcus)	41	1a	*MTOR* (c.6644C > T, p.Ser2215Phe)	2
13	FCDIIb	Left superior frontal gyrus	35, 35, 36	1a	*MTOR* (c.4370_4375dup, p. Glu1457_Asp1458dup)	3.7
14	FCDIIb	Left parietal (post central sulcus)	10, 18	4b	*MTOR* (c.6644C > T, p.Ser2215Phe)	3.5
15	FCDIIb	Right parieto-temporal (supra marginal gyrus and sup temporal gyrus)	18, 42	1a	*MTOR* (c.6644C > T, p.Ser2215Phe)	6.9
16	FCDIIb	Left medial frontal / cingulate sulcus	54	3a	*MTOR* (c.6644C > T, p.Ser2215Phe) *EIF4ENIF1* (c.1280-1G > A (splice site))	1.2 Germline (Candidate)

VAF: variant allele fraction. FCD: focal cortical dysplasia. VUS: variant of unknown significance. TSC: tuberous sclerosis. GG: ganglioglioma. HS: hippocampal sclerosis. NA: Not applicable (<12 months follow up from most recent surgery).

* FFPE only.

† Previously reported cases Case 1 (Lee et al., 2019), Case 3&4 (Bennett et al., 2022), Case 6 (Bizzotto et al., 2025), Case 8 (Leventer et al., 2015).

## Data Availability

Gene variants have been deposited in the publicly accessible Leiden Open Variation Database v3.0 (https://www.lovd.nl/), with a direct link provided upon request.

## Appendix A. Supplementary data

Supplementary data to this article can be found online at https://doi.org/10.1016/j.pnpbp.2026.111920.
