# Supplementary material for: Deep tissue sequencing improves genetic diagnostic yield in focal cortical dysplasia
Source: Prog Neuropsychopharmacol Biol Psychiatry. Author manuscript; Available in PMC 2026 Oct 10. (PMC13653638; doi:10.1016/j.pnpbp.2026.111920)
Supplement: Supplementary file 1 [file NIHMS2213706-supplement-1.docx]

**Supplementary Table 1.** Summary of findings in novel and established epilepsy genes, including unsolved cases. ACMG: American College of Medical Genetics and Genomics. gnomAD: Genome Aggregation Database. VAF: variant allele fraction. FCD: focal cortical dysplasia. VUS: variant of unknown significance. TSC: tuberous sclerosis. GG: ganglioglioma. HS: hippocampal sclerosis. NA: Not applicable (<12months follow up from most recent surgery)

| Case | Histopathology | Anatomical Location | Age at Surgery (years) | Surgical Outcome (Engel) | Gene Variant | ACMG | gnomAD (%) | Sequencing VAF (%) | NM Code | ENST Code | Tissue Sequencing Method | Solved |
| --- | --- | --- | --- | --- | --- | --- | --- | --- | --- | --- | --- | --- |
| 1^†^ | FCDIIa | Right temporal | 3 | 2a | *DEPDC5* (c.2390delA, p.Gln797Argfs*18) | Likely pathogenic | 0 | Germline | NM_001242896.3 | ENST00000400246.7 | WES 80x | Y |
| 2 | FCDIIa | Left frontal (cingulate gyrus) | 7 | 1a | *NPRL3* (c.393+4_393+7del) | Likely pathogenic | 0.001 | Germline | NM_001077350.3 | ENST00000611875.5 | WES 80x | Y |
| 3^†^ | FCDIIa | Left mesial frontal | 2 | 1a | *NPRL3* (c.48delG, p.Ser17Alafs*70) | Likely pathogenic | 0 | Germline | NM_001077350.3 | ENST00000611875.5 | WES 200x & 1000x* | Y |
| 4^†^ | FCDIIa | Right superior frontal gyrus (posteriorly) | 6 | 4a | *NPRL3* (c.48delG, p.Ser17Alafs*70)  *WNT2* (c.338C>T, p.Ala113Val) | Likely pathogenic  VUS | 0  0 | Germline  0.3 (Candidate) | NM_001077350.3  NM_003391.3 | ENST00000611875.5  ENST00000265441.8 | WES 1000x | Y |
| 5^†^ | FCDIIb | Left frontal pole | 49 | 1a | *DEPDC5* (c.4501C>T, p.Gln1501X) | Pathogenic | 0 | Germline | NM_001242896.3 | - | WES 200x  & Panel 2 | Y |
| 6 | FCDIIa | Left frontal | 0.5 | 4 | *MTOR* (c.6644C>T, p.Ser2215Phe) | Pathogenic | 0 | 1.2 | NM_004958.4 | ENST00000361445.9 | WES 400x &  Panel 1 | Y |
| 7^†^ | FCDIIa | Right hemispheric (extensive) | 0.8, 3.8 | 3a | *MTOR* (c.4487T>G, p.Trp1456Gly) | Likely pathogenic | 0 | 8.0 | NM_004958.4 | ENST00000361445.9 | WES 80x | Y |
| 8 | FCDIIb | Right temporal | 63 | 2a | *MTOR* (c.5395G>A, p.Glu1799Lys) | Pathogenic | 0 | 0.4 | NM_004958.4 | ENST00000361445.9 | Panel 2 | Y |
| 9 | FCDIIb | Left sup frontal gyrus | 24 | 1a | *MTOR* (c.4379T>C, p.Leu1460Pro) | Pathogenic | 0 | 0.7 | NM_004958.4 | ENST00000361445.9 | WES 400x  Panel 1 | Y |
| 10^*^ | FCDIIb | Left frontal (precentral sulcus) | 23 | NA (<12 months F/U) | *MTOR* (c.4376C>A, p.Ala1459Asp) | Pathogenic | 0 | 1.4 | NM_004958.4 | ENST00000361445.9 | Panel 1 | Y |
| 11 | FCDIIb | Right parietal (precuneus) | 25 | 1b | *MTOR* (c.5930C>A, p.Thr1977Lys) | Pathogenic | 0 | 5.4 | NM_004958.4 | ENST00000361445.9 | WES 200x | Y |
| 12 | FCDIIb | Right parietal (post central sulcus) | 41 | 1a | *MTOR* (c.6644C>T, p.Ser2215Phe) | Pathogenic | 0 | 2 | NM_004958.4 | ENST00000361445.9 | WES 400x | Y |
| 13 | FCDIIb | Left superior frontal gyrus | 35, 35, 36 | 1a | *MTOR* (c.4370_4375dup, p.Glu1457_Asp1458dup) | Likely pathogenic | 0 | 3.7 | NM_004958.4 | ENST00000361445.9 | WES 200x &  Panel 1 | Y |
| 14 | FCDIIb | Left parietal (post central sulcus) | 10, 18 | 4b | *MTOR* (c.6644C>T, p.Ser2215Phe) | Pathogenic | 0 | 3.5 | NM_004958.4 | ENST00000361445.9 | WES 80x | Y |
| 15 | FCDIIb | Right parieto-temporal (supra marginal gyrus and sup temporal gyrus) | 18, 42 | 1a | *MTOR* (c.6644C>T, p.Ser2215Phe) | Pathogenic | 0 | 6.9 | NM_004958.4 | ENST00000361445.9 | WES 400x | Y |
| 16 | FCDIIb | Left medial frontal (cingulate sulcus) | 54 | 3a | *MTOR* (c.6644C>T, p.Ser2215Phe)  *EIF4ENIF1* (c.1280-1G>A (splice site)) | Pathogenic  VUS | 0  0 | 1.2  Germline (Candidate) | NM_004958.4  NM_019843.4 | ENST00000361445.9  ENST00000330125.10 | WES 200x  Panel 2 | Y |
| 17 | FCDIIa | Right parietal (precuneus) | 35, 36 | 2b | *NPRL3* (c.275G>A, p.Arg92Gln) | VUS | 0.018 | Germline (Candidate) | NM_001077350.3 | ENST00000611875.5 | WES 200x | **N** |
| 18 | FCDIIb | Right frontal (cingulate sulcus) | 25 | 1a | *-* | - | - | - | - | - | WES 400x | N |
| 19 | FCDI | Right superior and middle frontal gyrus | 44 | 4a | - | - | - | - | - | - | WES 400x  Panel 1 | **N** |
| 20 | FCDI | Right medial frontal (anterior cingulate) | 2.5 | 1a | - | - | - | - | - | - | WES 200x | **N** |
| 21 | FCDIa | Left occipital | 33 | 4a | - | - | - | - | - | - | WES 400x | **N** |
| 22 | FCDIIa | Right post central sulcus | 27, 32 | 4a | *-* | - | - | - | - | - | WES 400x | **N** |
| 23 | FCDIIa | Left temporal | 5, 8 | 3 | - | - | - | - | - | - | Panel 1 | **N** |
| 24 | FCDIIb | Right ATL and right orbito-frontal, Right anterior cingulate | 38, 44 | 1a | - | - | - | - | - | - | WES 400x | **N** |
| 25 | FCDIIb | Left inferior frontal sulcus, Same region | 18, 19.5 | 1a | *-* | - | - | - | - | - | WES 400x | **N** |

^*^FFPE only

^†^Previously reported cases Case 1 (Lee et al., 2019), Case 3&4 (Bennett et al., 2022), Case 6 (Bizzotto., et al 2025), Case 8 (Leventer., et al 2015)

**Supplementary Table 2.** High-depth Austin Pathology panel sequencing gene list (n = 140)

| **MTOR** | **RAS** | | **Focal Epilepsy** | **Cancer** | | | | |
| --- | --- | --- | --- | --- | --- | --- | --- | --- |
| *AKT1* | *ALK* | *MAP2K1* | *ATP2A1* | *ACVR2A* | *CTNNB1* | *IDH2* | *NCOA4* | *SDC4* |
| *AKT3* | *BARD1* | *MAP3K3* | *GNA11* | *AIM2* | *DAXX* | *IL6ST* | *NR21* | *SDHA* |
| *DEPDC5* | *BRAF* | *MDM2* | *GNAQ* | *APC* | *DEPDC5* | *KBTBD4* | *NR24* | *SDHB* |
| *MTOR* | *BRCA1* | *MET* | *GRIN2C* | *ARID1A* | *DICER1* | *KDM6A* | *PALB2* | *SDHC* |
| *NPRL2* | *BRCA2* | *MYB* | *KLHL22* | *ASTE1* | *DROSHA* | *KEAP1* | *PBRM1* | *SDHD* |
| *NPRL3* | *BRIP1* | *MYC-N* | *SF3B1* | *ATM* | *EIF1AX* | *KIF5B* | *PDGFRA* | *SLC34A2* |
| *PIK3CA* | *CBL* | *NF1* | *SLC35A2* | *ATR* | *ELOC* | *KIT* | *PMS2* | *SMAD4* |
| *PIK3R1* | *CCND1* | *NF2* |  | *ATRX* | *EML4* | *KMT2C* | *POLE* | *SMARCA4* |
| *PTEN* | *CDK4* | *NRAS* |  | *BAP1* | *EPCAM* | *KMT2D* | *PPFIA4* | *TAF1B* |
| *RHEB* | *EGFR* | *PRKCA* |  | *BAT25* | *EZR* | *MARCKS* | *PRKAR1A* | *TEK* |
| *TSC1* | *ERBB2* | *PTPN11* |  | *BAT26* | *FANCL* | *MEN1* | *PRKD1* | *TERT* |
| *TSC2* | *FANCA* | *RAF1* |  | *CCDC6* | *FH* | *MITF* | *PTHLH* | *TFEB* |
|  | *FGFR1* | *RB1* |  | *CD74* | *FOXL2* | *MLH1* | *RAD51A* | *TGFBR2* |
|  | *FGFR2* | *RIT1* |  | *CDK12* | *GNAS* | *MONO27* | *RAD51B* |  |
|  | *FGFR3* | *ROS1* |  | *CDKN2A* | *H3-3A* | *MRE11* | *RAD51C* |  |
|  | *HRAS* | *SOS1* |  | *CDKN2B* | *H3C2* | *MSH2* | *RAD54L* |  |
|  | *KRAS* | *STK11* |  | *CHEK1* | *HNF1A* | *MSH6* | *RET* |  |
|  | *LZTR1* | *TP53* |  | *CHEK2* | *IDH1* | *NBN* | *RNF43* |  |

**Supplementary Table 3.** High-depth Panel 2sequencing gene list (n = 71)

**Supplementary Table 4.** *NPRL3* (NM_001077350.3): c.393+4_393+7del primer details. Primer set produces two products targeting two *NRPL3* transcripts; 465bp (includes 75bp exon 5), 390bp (excludes 75bp exon 5).

|  | Sequence (5’ > 3’) | Template Strand | Length | Tm | GC% |
| --- | --- | --- | --- | --- | --- |
| Forward | ACGGCGATTCCAGGTTTTCA | Plus | 20 | 60.25 | 50.00 |
| Reverse | ACGTGCACAGGCTGTCATAA | Minus | 20 | 59.97 | 50.00 |
| Product Length | 465 |  |  |  |  |

**Supplementary Table 5.** Validation of low-level somatic variants with ddPCR and amplicon sequencing

| **Case** | **Sequencing method** | **Sequencing VAF (%)** | **Validation method** | **Validation VAF (%)** | **Difference (pp)** |
| --- | --- | --- | --- | --- | --- |
| 11 | WES | 5.4 | ddPCR | 2.40 | −3.0 |
| 12 | WES | 2.0 | ddPCR | 2.80 | +0.8 |
| 14 | WES | 3.5 | ddPCR | 4.60 | +1.1 |
| 6 | Panel 1 | 1.2 | ddPCR | 1.20 | 0 |
| 10 | Panel 1 | 1.4 | ddPCR | 1.00 | −0.4 |
| 13 | Panel 1 | 3.7 | ddPCR | 4.30 | +0.6 |
| 9 | Panel 1 | 0.7 | ddPCR | 0.70 | 0 |
| 16 | Panel 2 | 1.2 | ddPCR | 1.6 | +0.4 |
| 8 | Panel 2 | 0.4 | Amplicon | 0.20 | −0.2 |

**Supplementary Figure 1.** RT-PCR analysis demonstrates aberrant splicing of *NPRL3* with preferential exclusion of exon 5 in brain-derived RNA from Case 2. A) Gel electrophoresis of RT-PCR products amplified with primers flanking exon 5 (Supplementary Table 3) shows the dominant *NPRL3* transcript excludes exon 5 in the variant carrier, unlike control brain tissue or control fibroblasts. B) Sanger sequencing of RT-PCR products confirms exon 5-containing transcripts are reduced in the variant carrier compared to controls.
